# Novel Sol-Gel Synthesis Route for Ce- and V-Doped Ba_0.85_Ca_0.15_Ti_0.9_Zr_0.1_O_3_ Piezoceramics

**DOI:** 10.3390/ma17133228

**Published:** 2024-07-01

**Authors:** Larissa S. Marques, Michelle Weichelt, Michel Kuhfuß, Carlos R. Rambo, Tobias Fey

**Affiliations:** 1 Graduate Program on Materials Science and Engineering, Federal University of Santa Catarina, Florianópolis 88040-900, Brazil; larissa.silva.m@posgrad.ufsc.br (L.S.M.); carlos.rambo@ufsc.br (C.R.R.); 2 Department of Material Science and Engineering, Institute of Glass and Ceramics, Friedrich-Alexander Universität Erlangen-Nürnberg, Martensstr. 5, 91058 Erlangen, Germany; michelle.mw.weichelt@fau.de (M.W.); michel.kuhfuss@fau.de (M.K.); 3 NITech Doctoral Global Academy, Nagoya Institute of Technology, Gokiso-cho, Showa-ku, Nagoya 466-8555, Japan

**Keywords:** lead-free piezoceramics, Ba_0.85_Ca_0.15_Ti_0.9_Zr_0.1_O_3_, (BCZT), doping, vanadium, cerium, sol-gel

## Abstract

To meet the current demand for lead-free piezoelectric ceramics, a novel sol-gel synthesis route is presented for the preparation of Ba_0.85_Ca_0.15_Ti_0.9_Zr_0.1_O_3_ doped with cerium (Ce = 0, 0.01, and 0.02 mol%) and vanadium (V = 0, 0.3, and 0.4 mol%). X-ray diffraction patterns reveal the formation of a perovskite phase (space group *P*4*mm*) for all samples after calcination at 800 °C and sintering at 1250, 1350, and 1450 °C, where it is proposed that both dopants occupy the B site. Sintering studies show that V doping allows the sintering temperature to be reduced to at least 1250 °C. Undoped BCZT samples sintered at the same temperature show reduced functional properties compared to V-doped samples, i.e., d_33_ values increase by an order of magnitude with doping. The dissipation factor tan δ decreases with increasing sintering temperature for all doping concentrations, while the Curie temperature T_C_ increases for all V-doped samples, reaching 120 °C for high-concentration co-doped samples. All results indicate that vanadium doping can facilitate the processing of BCZT at lower sintering temperatures without compromising performance while promoting thermal property stability.

## 1. Introduction

Piezoelectric materials have the ability to convert mechanical stress into an electrical charge as well as conversely produce a mechanical output in response to an applied electric field [1,2]. This direct link between electrical and mechanical stimuli makes them important in numerous applications, such as piezoelectric energy harvesting systems for self-powered sensors, transducers in medical applications, actuators in vibration suppression mechanisms, underwater microphones and speakers, and many more [2,3,4,5,6,7,8]. Understandably, perovskite piezoelectrics are of particular interest, especially lead-free compositions, since their discovery due to their high piezoelectric and dielectric coefficients as well as excellent electromechanical coupling factors. In addition to this, tunability of the electromechanical properties through doping is important for tailoring the functional properties to the application. Despite their excellent electromechanical properties, lead-containing compositions, typically based on PbZrO_3_–PbTiO_3_ solid solutions (PZT), pose environmental and health concerns [9,10,11,12]. Consequently, considerable research has focused on developing lead-free alternatives [9].

In particular, the 1 − x(Ba_0.7_Ca_0.3_TiO_3_) − x(BaZr_0.2_Ti_0.8_O_3_) system has attracted interest due to the excellent piezoelectric properties discovered near its polymorphic phase transition (PPB) [13]. This boundary is particularly important because it marks the transition zone between the tetragonal (Ca-rich) and rhombohedral (Zr-rich) phases, which is crucial for the enhanced piezoelectric and ferroelectric properties of the material [14]. The presence of the PPB allows for an unusual flexibility in the structure of the material, allowing for an increased sensitivity to external stimuli such as electric fields and temperature changes [15]. This sensitivity is due to the inherent instability at the interface, which facilitates polarization and increases the electromechanical coupling coefficient of the material.

This is shown by a large number of studies on the doping, processing, synthesis, and defect engineering of BCZT [16,17,18,19,20,21,22,23,24,25,26,27]. Among these key research topics, perovskite doping attracts special attention because it allows for the development of a range of functional properties to suit the application requirements. Despite the importance, doping in lead-free ferroelectrics remains poorly understood, and many of the lessons from lead-containing materials cannot be easily transferred to lead-free compositions [28,29]. Doping can be carried out in both A and B sites by isovalent and aliovalent dopants and has the most diverse effects on important technological properties, such as the phase boundaries as well as the small and large signal electromechanical properties. In addition, doping has a direct effect on processing parameters, such as sintering temperature. Although dense BCZT ceramics can be obtained by sintering at 1300 °C, higher temperatures, around 1475 °C, are required to achieve a microstructure, i.e., an adequate grain size, that allows for optimal functional properties [30]. Elements such as Ce, especially using cerium oxide as a precursor in solid-state synthesis processes, and V have been found to individually reduce the sintering temperature of BCZT. However, little is known about their simultaneous effects on the structure and, in particular, on the dielectric and piezoelectric properties [30,31,32,33,34,35]. For example, it is not known whether these dopants will have a synergistic effect in lowering the sintering temperature when introduced simultaneously into the BCZT structure. Rather than adding the dopant oxides to the calcined BCZT powder, which is the prevalent doping practice, an alternative method is proposed. The dopant precursors become a part of the sol-gel BCZT process. This allows for exclusive analysis of the role of V and Ce elements in the BCZT structure and the corresponding effects on its properties.

In this work, a novel sol-gel synthesis route using both V and Ce precursors as part of the sol-gel synthesis process, adapting the sol-gel route described by Köllner et al. [36], is presented. This synthesis method was chosen for its compatibility with doping regarding easy incorporation, uniform doping, and exceptional process control to analyze the effects of these dopants in BCZT without the influence of possible microstructural features that may result from these reagents when added later in the process [37]. The doping concentration range was chosen based on previously published works around optimum values presented for both dopants [30,31,32,33,34,35]. In addition, this novel approach to doping BCZT with V and Ce (and of course other possible elements) allows for a simpler process, since there is no need for an additional step regarding their proper homogenization in the calcined BCZT powder. In order to reveal the effects of Ce and V doping on the microstructure and piezoelectric and ferroelectric properties of BCZT, we carried out structural, microstructural, dielectric, and piezoelectric investigations to determine the potential of Ce and V in facilitating the sol-gel synthesis process of BCZT and its effects.

## 2. Materials and Methods

### 2.1. Synthesis

**Figure 1:**
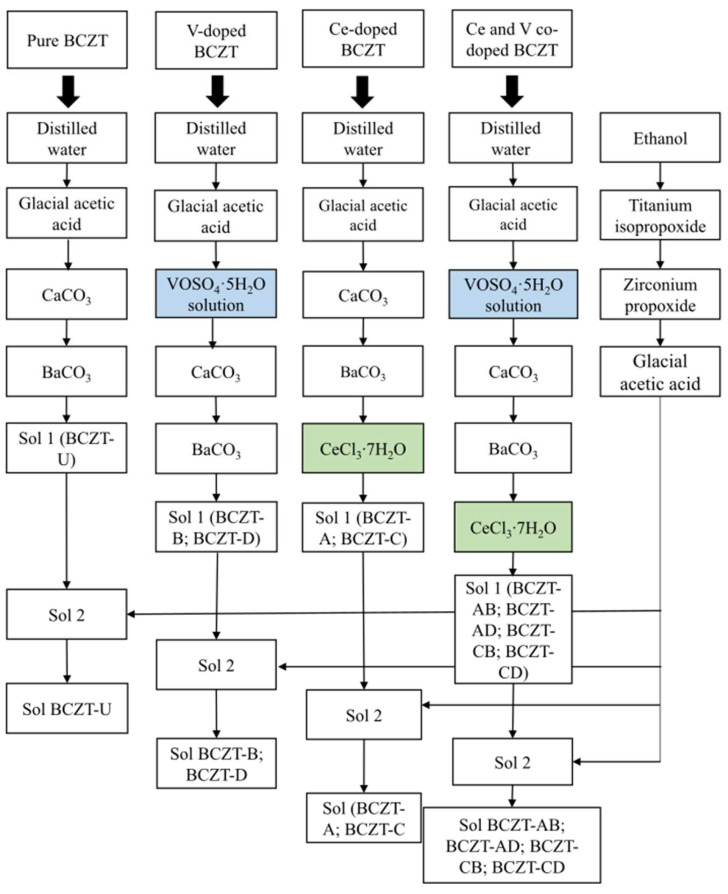
Schematic representation of the synthesis process for pure BCZT, V-doped BCZT, Ce-doped BCZT, and Ce and V co-doped BCZT sols.

Pure BCZT (BCZT-U), Ce-doped BCZT (Ce = 0.01 and 0.02 mol%, compositions BCZT-C and BCZT-A, respectively), V-doped BCZT (V = 0.3 and 0.4 mol%, compositions BCZT-D and BCZT-B, respectively), and V and Ce co-doped BCZT (BCZT-AB, BCZT-AD, BCZT-CB, and BCZT-CD compositions) were synthesized as shown in Figure 1. Novel sol-gel routes were created by adapting the process described by Köllner et al. regarding the addition of Ce and V dopants through chloride and sulfate, respectively [36]. The following reagents were used in the synthesis process: 100% glacial acetic acid (Supelco. Darmstadt, Germany), CaCO_3_ (99%, Sigma Aldrich, Darmstadt, Germany), BaCO_3_ (99%, Alfa Aesar, Karlsruhe, Germany), ethanol (99%, Euro denatured VWR Chemicals, Ismaning, Germany), titanium isopropoxide IV (Sigma Aldrich, 97%), zirconium IV n-propoxide 70% *w*/*w* in n-propanol (Alfa Aesar), CeCl_3_·7H_2_O (Alfa Aesar, 99%), and vanadyl sulfate pentahydrate (Technical grade, VWR chemicals).

The synthesis was divided into two parts, preparing solutions 1 and 2, following a procedure similar to that described by Köllner et al. [36]. BCZT-U was prepared by sequentially adding calcium carbonate (0.027 mol) and barium carbonate (0.15 mol) to a solution of distilled water (9.7 mol) and acetic acid (1.2 mol) under continuous stirring to create solution 1. Solution 2 was prepared by sequentially adding titanium isopropoxide (0.16 mol) and zirconium n-propoxide (0.018 mol) to ethanol (1.1 mol) with continuous stirring, followed by the addition of acetic acid (1.9 mol). Solution 2 was then poured into solution 1 to obtain the desired Ba_0.85_Ca_0.15_Ti_0.9_Zr_0.1_O_3_ composition. For ageing, the sol was kept under stirring for 24 h. To synthesize the Ce-doped BCZT compositions, (BCZT-A and BCZT-C), the appropriate amounts of Ce chloride (0.18 mmol for the BCZT-A composition and 0.9 mmol for the BCZT-C composition) were added to solution 1. All other synthesis steps were the same as those previously described for BCZT-U (see Figure 1 for a better understanding). For the synthesis of the V-doped BCZT ceramics (BCZT-B and BCZT-D), the appropriate amounts of vanadyl sulfate (3.53 mmol for the BCZT-B composition and 2.77 mmol for the BCZT-D composition) were dissolved in distilled water (0.3 mol for the BCZT-B composition and 0.24 mol for the BCZT-D composition) and added to solution A. The remaining steps were identical to those for the synthesis of BCZT-U sol, as can be seen in Figure 1. For the co-doped compositions (BCZT-AB, BCZT-AD, BCZT-CB, and BCZT-CD), the same steps for the addition of dopants were followed as for the separate preparation of Ce-doped and V-doped ceramics. All other synthesis steps were the same as for BCZT-U (see Figure 1). Sample labeling is given in Table 1, which contains the doping content of all samples and their abbreviations.

### 2.2. Sample Preparation

After aging, the sols were gelled using the following thermal program: initially, heating at 190 °C for 10 min, followed by 200 °C for 20 min, then increasing by 15 °C and maintaining the temperature until complete gelation was achieved. Drying of the gels was performed in a muffle furnace for 24 h at 250 °C to obtain xerogels, followed by calcination in air at 800 °C, a 5 K/min heating rate, and a 5 h dwell time to promote crystallization. Calcined powders were prepared for sintering by grinding for 96 h in ethanol using 5 mm zirconia beads as the grinding medium. The homogeneous powders were then dried at 90 °C for 4 h over a heating plate.

For sintering studies, the calcined powders were mixed with a binder, 8 wt% PVA (polyvinyl alcohol) solution, and homogenized using a porcelain mortar and pestle. The powders were compacted in a uniaxial press at 125 MPa for 3 min using a steel die with an internal diameter of 10.2 mm as a mold. After binder removal (1 h at 500 °C at a heating rate of 3 K/min), sintering was performed at three different temperatures, 1250, 1350, and 1450 °C, for 4 h in a program-controlled furnace with a heating rate of 4 K/min. After sintering, the surfaces of the pellets were made plane-parallel by grinding them with SiC paper, followed by a final grinding step using a surface grinder (Jung, JF415-S) to a thickness of 0.8 mm. To relieve stresses caused by the grinding process, the samples were annealed at 500 °C for 2 h at a heating rate of 2 K/min and cooled at an uncontrolled rate.

### 2.3. Characterization Methods

X-ray diffraction measurements of the calcined and sintered samples were performed in the reflection mode using a Bruker diffractometer (D8-Advance) (Bruker, Billerica, MA, USA) equipped with a copper source, a step size of 0.02°, a scan step time of 1 s, voltage of 40 kV, current of 25 mA, and a measuring range of 15 to 90°. Metallic contacts were made by applying silver paint (G302-Leitsilber) to both sides of the sintered samples and drying them naturally for 1 h before polishing and performing dielectric and piezoelectric measurements.

Micrographs of sintered samples were obtained using a JEOL JSM-IT500HR Emission Scanning Electron Microscope (SEM) (JEOL Ltd., Tokyo, Japan) and analyzed with ImageJ (version 1.54j) [38].

Dielectric measurements were performed using an LCR meter (Keysight E4980AL, Keysight, Santa Rosa, CA, USA) coupled to a program-controlled furnace (Nabertherm LE 4/11/3216, Nabertherm GmbH, Lilienthal, Germany) to obtain permittivity (ε_r_) and a dissipation factor at 1 kHz as a function of temperature from room temperature to 200 °C at a heating rate of 2 K/min.

The piezoelectric coefficient (d_33_) measurements were performed on electrically poled samples at room temperature using the high-precision PiezoMeter PM300 system (110 Hz frequency and 10 N griping force as measuring settings) (Piezotest Ltd., London, UK). Poling was performed by applying an electric field of 3 kV/mm for 30 min at room temperature, whereby the sample was inserted in a silicone oil bath (Wacker Silicone Fluid AP 150, Wacker, Munich, Germany).

The density of the sintered samples was measured using the Archimedes method using an analytical balance (Mettler Toledo AG204, Mettler Toledo, Greifensee, Switzerland).

## 3. Results and Discussions

**Figure 2:**
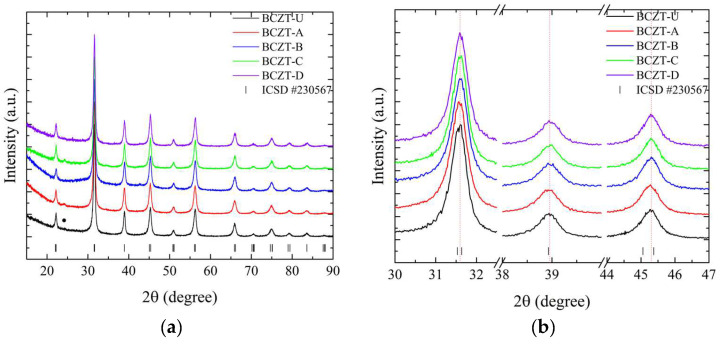

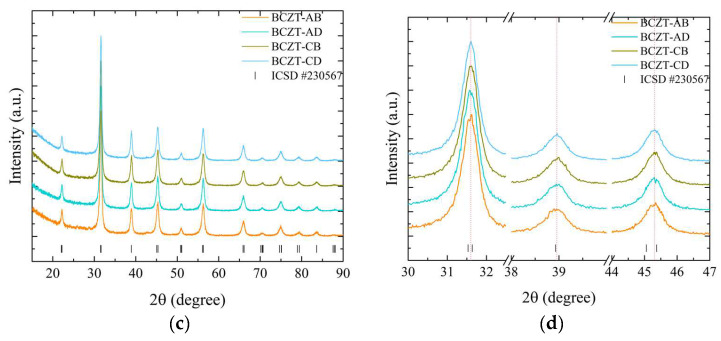
X-ray diffraction patterns of calcinated samples at 800 °C for 5 h from (**a**) 15 to 90° for undoped and doped samples; (**b**) 30 to 47° for undoped and doped samples; (**c**) 15 to 90° for co-doped samples; and (**d**) 30 to 47° for co-doped samples.

As demonstrated in Figure 2, the employed synthesis route is effective in producing the targeted cerium-doped, vanadium-doped, and co-doped (Ce and V) BCZT ceramics within the ferroelectric tetragonal phase. For a more detailed overview, please refer to Table 1, which includes the labeling, doping content, and identification of the samples. The diffraction patterns obtained demonstrate that the heat treatment at 800 °C was successful in promoting crystallization, with no amorphous phase identified within the equipment’s detection limit. For the composition range studied, it was observed that BCZT’s perovskite structure was maintained for doped and co-doped samples, and no composition-induced phase transition was observed. With the ICSD (Inorganic Crystal Structure Database) CIF (Crystallographic Information File) collection code number 230567, all diffraction peaks can be indexed to the tetragonal *P*4*mm* space group [25,39]. Calcinated Ce-doped samples exhibited a minor secondary phase at 24.3° marked with a star in Figure 2.

The peak shifts observed in the doping experiments indicate that Ce occupies distinct crystallographic sites depending on concentration, while V occupies only the B site. Furthermore, both elements were successfully introduced into BCZT’s structure to form a solid solution. For the low-concentration Ce-doped BCZT sample (BCZT-C), the diffraction peaks shift to higher angles, indicating a corresponding shift to smaller lattice parameters that occur when Ce occupies the barium site (A site). This phenomenon can be observed in Figure 2b. This shift occurs due to the substitution of the larger Ba^2+^ ion (ionic radius 1.60 nm) by the smaller Ce^3+^ (1.01 nm) or Ce^4+^ (0.87 nm) ions [40]. Conversely, diffraction peaks shift to lower angles when the Ce concentration is higher (BCZT-A), which indicates that cerium occupies the B site. This is consistent with findings confirming that the Ce ion can occupy both the A and B crystallographic sites, depending on the oxidation state of cerium.

The Ce^3+^ ion (ionic radius, 1.01 nm) occupies the A site as a donor dopant and Ce^4+^ (ionic radius, 0.87 nm) the B site as an isovalent dopant. When Ce^4+^ substitutes either Ti^4+^ (ionic radius 0.605 nm) or Zr^4+^ (0.72 nm) atoms, the lattice parameters increase, and the peaks shift to lower angles [41,42]. In the case of V-doped BCZT samples, the increase in V concentration results in the diffraction peaks shifting consistently to higher diffraction angles due to the smaller lattice parameters, as illustrated in Figure 2b. In this instance, the V ion (either V^4+^ or V^5+^, with a radius of 0.59 or 0.46 nm, respectively) replaces the larger Ti^4+^ or Zr^4+^ ions, as previously reported elsewhere [34,42]. As the doping concentration increases when co-doping from BCZT-CD to BCZT-AB, the peaks present a continuous subtle shift to higher angles, indicating a continuous reduction of the lattice parameters. This is consistent with the substitutions discussed. This evidence demonstrates the successful integration of these ions into the BCZT structure. However, further structural analysis is required to fully comprehend the manner in which these ions occupy the A and B sites when competing for the same site.

**Figure 3:**
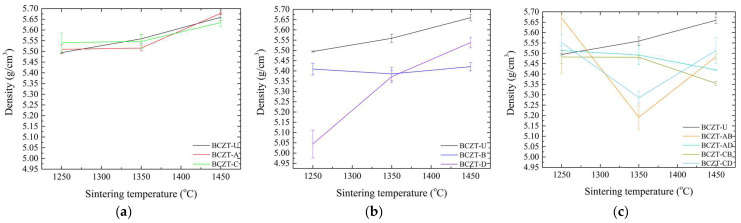
Density values as a function of sintering temperature for (**a**) Ce-doped BCZT samples; (**b**) V-doped BCZT samples; and (**c**) Ce and V co-doped BCZT samples.

Sintering is a crucial process for obtaining fully dense samples, as it directly impacts the dielectric, ferroelectric, and piezoelectric properties. These properties are of particular importance in the context of piezoceramics, as they influence overall performance. To ascertain that adequate densification was achieved, density values were calculated for all compositions and are presented as a function of sintering temperature, as seen in Figure 3.

Figure 3 presents a comparison of the density values of doped and co-doped BCZT ceramics with those of undoped BCZT ceramics. It can be observed that the density of BCZT-U varies almost linearly with the sintering temperature. However, the doping process alters this behavior according to the concentration and dopants present. The theoretical density of BCZT-U is 5.772 g/cm^3^. Samples sintered at 1450 °C exhibited a relative density of 98.0%, which is consistent with previously reported values [25,43]. As the concentration of Ce in the doped samples was low, the density values of the Ce-doped samples were similar to those of BCZT-U throughout the sintering temperature range. Furthermore, the form in which density varies as a function of sintering temperature was similar for both the Ce-doped and BCZT-U samples (Figure 3a).

Conversely, V doping was carried out at higher concentrations, which resulted in greater impact on the density values of V-doped BCZT and co-doped BCZT samples, as illustrated in Figure 3b,c. For high vanadium concentration samples, density remained almost unchanged within the sintering temperature range studied, indicating that full densification can be achieved at a lower sintering temperature. This phenomenon was particularly pronounced at the lowest sintering temperature investigated, 1250 °C. High-concentration V co-doped samples exhibited a considerably smaller gain in density when the sintering temperature increased from 1250 to 1350 °C, a range in which density gain is critical for undoped BCZT and Ce-doped BCZT samples. The reduction in density observed for co-doped samples when the sintering temperature increased had already been observed in Ce-doped samples [30]. Here, when co-doping, a similar phenomenon can be seen, especially when sintering increased from 1350 to 1450 °C.

**Figure 4:**
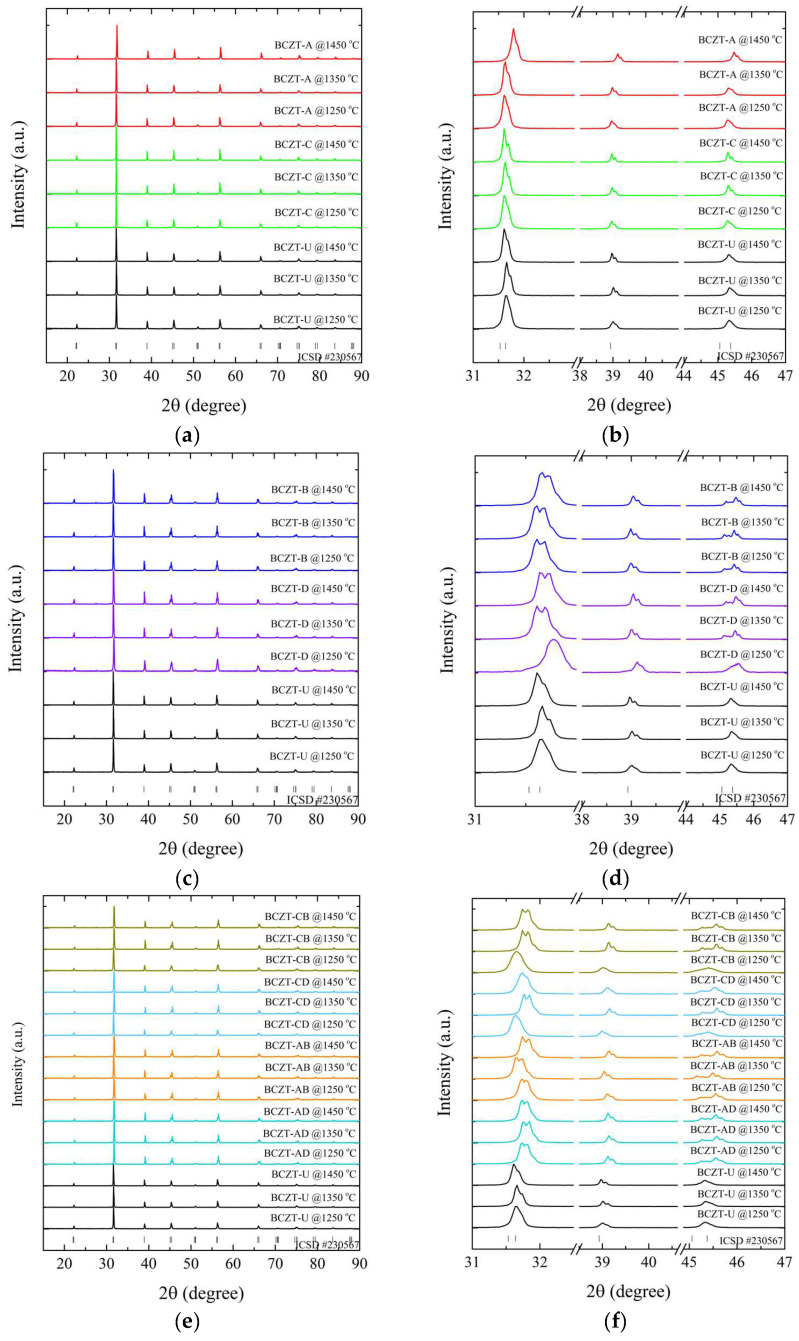
X-ray diffraction patterns of sintered samples regarding (**a**) Ce-doped samples compared to undoped BCZT samples, 15°–90°; (**b**) magnified peaks of Ce-doped samples compared to BCZT samples, 31°–47°; (**c**) V-doped samples compared to undoped BCZT samples, 15°–90°; (**d**) magnified peaks of V-doped samples compared to undoped BCZT samples, 15°–90°; (**e**) co-doped samples compared to BCZT samples, 15°–90°; and (**f**) magnified peaks of co-doped samples, 31°–47°.

X-ray diffraction measurements at room temperature were performed to determine the role of Ce and V dopants on the crystal structure of the sintered samples, as depicted in Figure 4.

The results of the XRD patterns shown in Figure 4a,c,e demonstrate that all BCZT samples display the perovskite structure for all investigated sintering temperatures, without the formation of secondary phases of Ce- or V-rich oxides. The diffraction patterns exhibited distinctive, well-defined sharp peaks, indicating the homogeneity and formation of solid solutions, comparable to the observations made in the calcined samples, namely the successful introduction of V and Ce into the BCZT lattice. The thermal treatments did not induce any observable secondary phases; the minor secondary phase observed in the Ce-doped calcinated samples was absent in the sintered samples.

The diffraction patterns were analyzed to understand the impact of sintering temperature on the samples’ structural properties. It was observed that an increase in temperature caused a corresponding sharpening of the diffraction peaks for all samples. However, the lattice parameters varied depending on the concentration of the dopant. This narrowing of the peaks indicated a reduction in strain, which was accompanied by an increase in crystallite size. This increase in crystallite size had a direct impact on the properties of the samples [44,45].

**Figure 5:**
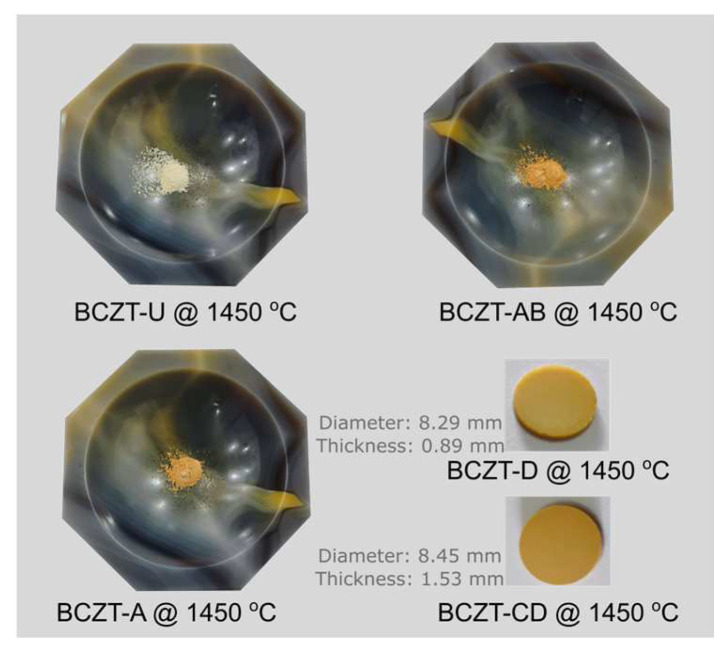
Photographs of selected sintered samples in pellet and powder form.

A comparison of sintered Ce-doped BCZT to undoped BCZT reveals that with an increase in Ce concentration, diffraction peaks shift to smaller diffraction angles, in contrast to what occurs in some calcinated samples (Figure 4b). Upon sintering, Ce occupies the B site in BCZT’s structure. This phenomenon occurred in all Ce-doped samples, with the exception of the BCZT-A sample sintered at 1450 °C. When V was considered, a constant peak shift to higher diffraction angles was observed with the increase of vanadium concentration. This same phenomenon was previously observed in the calcinated samples caused by the substitution of Zr and Ti by V in the B site, as illustrated in Figure 4c,d. In the case of the co-doped samples, there was a shift to higher diffraction angles in comparison to BCZT-U. This was due to the introduction of the dopants into BCZT’s lattice, which indicates that they experienced a reduction in the introduction of smaller Ce and V atoms replacing the bigger Zr and Ti atom results in a reduction in the lattice distance, as illustrated in Figure 4e,f. This is consistent with the peak shifts observed in the V- and Ce-doped BCZT samples, which have already been discussed. Pictures of selected sintered samples can be seen in Figure 5. Powder samples were used for XRD measurements.

**Figure 6:**
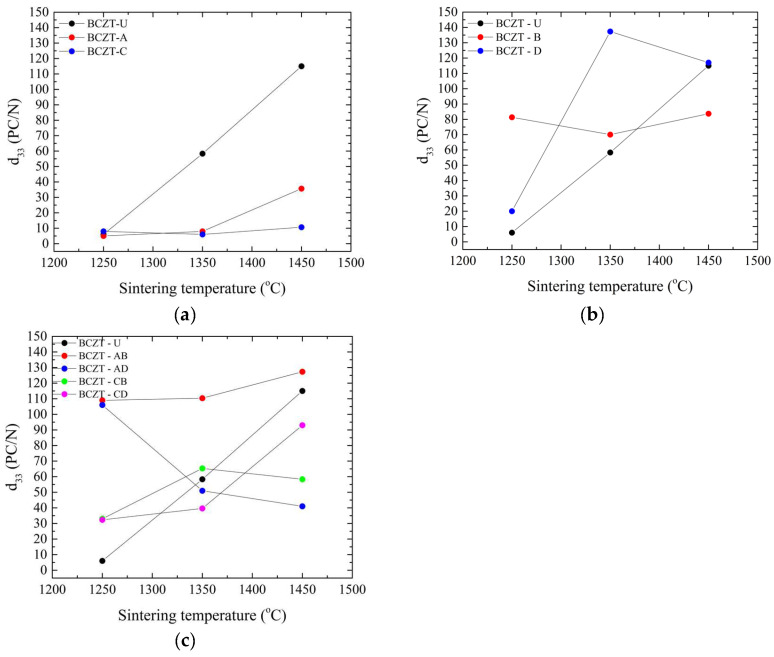
Piezoelectric measurements of all sintered samples as a function of sintering temperature: (**a**) d_33_ values for Ce-doped samples; (**b**) d_33_ values for V-doped samples; and (**c**) d_33_ values for co-doped samples.

The impact of these structural changes induced by Ce and V doping can be observed in the functional properties measured. This can be seen in the d_33_ values of polarized samples, as shown in Figure 6.

In the composition range studied, Ce doping significantly reduced the piezoelectric properties of BCZT, as can be seen in Figure 6a. As expected, it improved with the increase in sintering temperature. This behavior is consistent with the density values obtained; it is known that densification in terms of Ce doping larger grains (up to 30 µm) improves piezoelectric performance in BCZT [30] in contrast to undoped BCZT, where a grain size up to 10 µm improves the properties [46]. V doping, Figure 6b,c, on the other hand, had a more varied impact on BCZT’s piezoelectric properties compared to the Ce-doped samples. V-doped BCZT samples presented better performance, even when subjected to low sintering temperatures, such as at 1250 °C. When sintered at 1350 °C, the BCZT-D sample showed the greatest increase in d_33_ amongst all samples, potentially due to the link between grain size and sintering temperature. It is apparent that V is responsible, as all co-doped samples also outperformed undoped and Ce-doped samples when sintered at 1250 °C, as did BCZT-AB and BCZT-CB when sintered at 1350 °C, both with higher V concentrations. After increasing the sintering temperature, such as to 1450 °C, BCZT-D presents slightly better piezoelectric performance than undoped BCZT.

**Figure 7:**
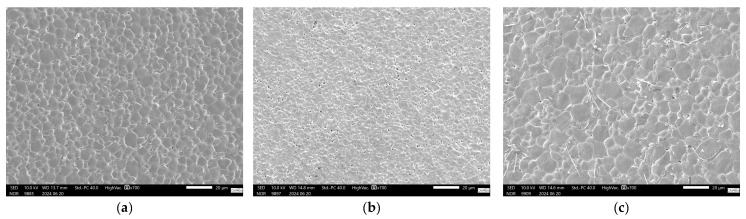
SEM pictures of sintered samples: (**a**) BCZT-U sample sintered at 1450 °C; (**b**) BCZT-A sample sintered at 1350 °C; (**c**) BCZT-AB sample sintered at 1350 °C.

For co-doped samples, Figure 6c, it is clear that the V addition is responsible for a better piezoelectric performance in the compositional range investigated, as all co-doped samples outperformed undoped samples when sintered at the lowest sintering temperature investigated, 1250 °C. When the sintering temperature was increased to 1350 °C, the V-rich samples BCZT-AB and BCZT-CB also outperformed the undoped samples. However, at the highest sintering temperature, the piezoelectric properties of the co-doped samples decreased, which may be related to the previously mentioned decrease in density at higher sintering temperatures. SEM micrographs are shown in Figure 7, which helps shed light on the reason behind the poor performance of Ce-doped samples in comparison to undoped BCZT, Figure 7a. Small grains and increased porosity are evident, as indicated by a 4.63 µm mean grain size (SD = 1.72) in 7b compared to a grain size of 7.6 µm (SD = 2.6) in 7a. On the other hand, co-doped BCZT samples, even at low temperatures, presented coarser grains of 10.05 µm (SD = 3.4), which was prompted by the V-doping, as shown in Figure 7c.

**Figure 8:**
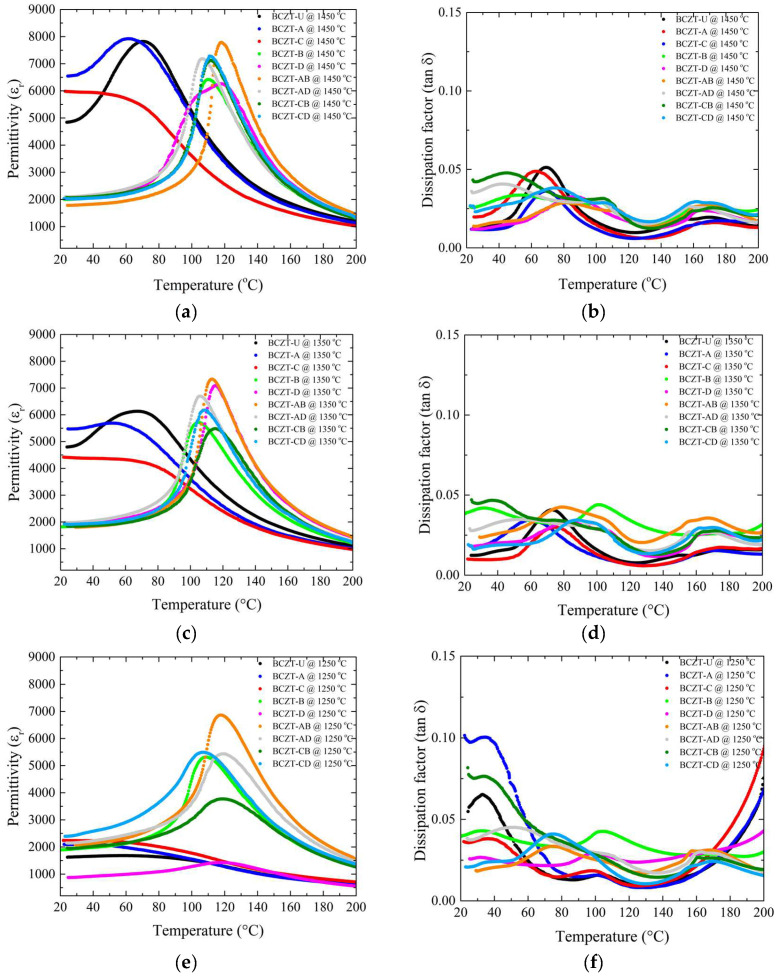
Dielectric and piezoelectric measurements of all sintered samples as a function of sintering temperature: (**a**) d_33_ values for Ce-doped samples; (**b**) capacitance values for Ce-doped samples; (**c**) d_33_ values for V-doped samples; (**d**) capacitance values for V-doped samples; (**e**) d_33_ values for co-doped samples; (**f**) capacitance values for co-doped samples.

The role of Ce and V dopants on the temperature-dependent dielectric response is shown in Figure 8. As the sintering temperature was increased from 1250 to 1350 °C, all samples experienced an increase in the maximum dielectric permittivity. However, an important aspect is that the high-concentration V-doped samples showed remarkably high permittivity values even at the lowest sintering temperature, so that the increase, although occurring, was more subtle (Figure 8a,c,e). The room-temperature permittivity was stable for V-doped samples at all sintering temperatures used; however, it increased significantly for Ce-doped and undoped BCZT samples when the sintering temperature was 1350 °C or higher. Densification and thermally activated microstructural changes associated with appropriate grain size were most likely responsible for this overall increase in permittivity. This also explains why the V-doped samples did not show such a significant increase in permittivity over the same temperature range as the Ce-doped and BCZT-U samples, as their density values were stable over the temperature range studied. As the sintering temperature was further increased, this phenomenon became more pronounced, as the permittivity of the Ce-doped and undoped BCZT samples increased with the densification, while the density of the V-doped samples had already reached a maximum when sintered at 1250 °C. As a result, the permittivity of the V-doped samples did not increase with the sintering temperature. Consequently, the dielectric permittivity did not increase, or the increase was less significant, because there was no further densification, and increased grain size did not seem to have a positive effect here. There was no advantage in increasing the sintering temperature to 1450 °C when V-doped samples were considered.

Sintering at 1350 °C showed dielectric loss values for all samples in the compositional range studied (Figure 8b,d,f). Sintering at 1250 °C was not as efficient for all the samples, but most of the samples showed losses inferior to those of the BCZT-U samples, with the exception of the BCZT-A and BCZT-CB samples, which led to the conclusion that, overall, the V and Ce co-doping of BCZT is beneficial for reducing the dielectric losses and that the samples showed adequate losses even at the lowest sintering temperature. Increasing the sintering temperature to 1450 °C would not help to further reduce the dielectric loss values. It is also worth noting that the dissipation did not vary significantly with increasing sintering temperature.

Another notable finding is that V is responsible for a significant increase in the Curie temperature, exceeding the values typically observed in undoped BCZT and Ce-doped BCZT samples [17,30,41]. In the sintering temperature range investigated, it can be seen that the V-doped samples had higher Curie temperatures than the non-doped or Ce-doped BCZTs.

The overall effect of Ce and V doping on the dielectric, ferroelectric, and piezoelectric properties of BCZT can be better understood by referring to Table 2. V doping has a notable effect on densification, as we achieved full densification by sintering at temperatures 200 °C lower than usual for regular BCZT. For Ce-doped and undoped BCZT, full densification could only be achieved when the sintering temperature reached 1450 °C, which had a positive effect on the overall properties. However, the V-doped samples, BCZT-D and BCZT-B, did not require such high temperatures, and peak performance was found at lower sintering temperatures. Due to the V effect on the crystal structure in BCZT, at the remarkably low sintering temperature of 1250 °C, we obtained unusually high values of relative permittivity, 5719.596, and d_33_ values more than 10 times higher than those of undoped BCZT. This effect was observed not only in V-doped samples but also in co-doped samples, and the addition of Ce did not reduce this effect for co-doping. The same can be seen for the Curie temperature, which was greatly increased when V was added to BCZT, going from 71 to almost 120 °C for BCZT-AB and BCZT-AD samples, even when the sintering temperature was as low as 1250 °C. In comparison to other lead-free and lead-based materials, refer to Table 3, the V-doped BCZT samples prepared in this work present properties compatible with conventional lead-free compositions, although there is still possibility to improve the piezo response with polarization optimization and fine-tuning of V concentration to obtain improved T_C_ values [47].

## 4. Conclusions

The doping and co-doping of BCZT with V and Ce can be realized exclusively by the sol-gel method, without the need for additional oxide steps. Through a novel straightforward sol-gel route followed by calcination at 800 °C, it is possible to obtain the tetragonal perovskites for the entire compositional range studied. Sintering at different temperatures, 1250, 1350 and 1450 °C, does not induce the nucleation of secondary phases or any phase transition. By analyzing the peak shifts in the diffraction patterns of all sintered samples, we were able to determine that Ce and V occupy the B site in the ABO_3_ structure, substituting for Ti and Zr atoms. Sintering studies have shown that the incorporation of V is beneficial for lowering the sintering temperature of BCZT. This reduction can be substantial, reaching at least a delta of 200 K compared to undoped BCZT. This reduction means a corresponding significant reduction in the energy required to process BCZT, which is a significant gain in terms of a more environmentally friendly process. Piezoelectric coefficient d_33_ measurements confirmed that Ce has a detrimental effect on the piezoelectric properties of BCZT, while V can improve them, especially when considering samples sintered at 1250 and 1350 °C. High values of maximum dielectric permittivity can also be obtained when BCZT samples are doped with 0.3–0.4 mol% V, even when sintered at low temperatures. In addition, V proves to be a candidate for increasing the Curie temperature of BCZT, with values almost 50 °C higher than those of undoped BCZT, which is interesting from the point of view of thermal stability, also seen in the room temperature values of the dielectric constant. Ce and V doping are responsible for the reduction of the total dielectric loss in BCZT. Considering these factors, vanadium is a promising candidate for facilitating the processing of BCZT without compromising performance. Thus, there is great potential in further in-depth structural investigation of V-doped BCZT aligned to additional microstructural analysis, ferroelectric characterization, and polarization optimization to obtain the best possible piezoelectric response.

## Figures and Tables

**Table 1:** Sample labeling, BCZT-X with X = abbreviation.

Abbreviation	Meaning
U	Undoped samples
A	Samples doped with 0.02 mol% of Ce
B	Samples doped with 0.4 mol% of V
C	Samples doped with 0.01 mol% of Ce
D	Samples doped with 0.3 mol% of V
AB	Samples doped with 0.02 mol% of Ce and 0.4 mol% of V
AD	Samples doped with 0.02 mol% of Ce and 0.3 mol% of V
CB	Samples doped with 0.01 mol% of Ce and 0.4 mol% of V
CD	Samples doped with 0.01 mol% of Ce and 0.3 mol% of V

**Table 2:** BCZT undoped, doped, and co-doped summary of important properties.

Sample	Sintering Temperature [°C]	Archimedes Density [g/cm^3^]	Standard Deviation	Maximum Permittivity [1 kHz]	d_33_ [PC/N]	T_C_ [at 1 kHz]	tan δ [at 1 kHz, RT]
BCZT-U	1250	5.494	0.003	1682.634	6	60.013	0.055
	1350	5.559	0.020	6131.59	58.333	68.585	0.012
	1450	5.659	0.016	7819.863	115	71.062	0.012
BCZT-A	1250	5.510	0.021	2084.624	5	45.031	0.099
	1350	5.516	0.013	5402.391	8	68.097	0.018
	1450	5.678	0.005	7799.113	35.67	68.598	0.020
BCZT-B	1250	5.409	0.028	5314.521	81.333	109.069	0.041
	1350	5.386	0.033	5719.596	70	105.145	0.040
	1450	5.421	0.021	6419.387	83.667	109.974	0.026
BCZT-C	1250	5.542	0.044	2148.203	8	64.534	0.036
	1350	5.546	0.033	4082.467	6	78.631	0.010
	1450	5.635	0.019	5434.032	10.667	70.083	0.012
BCZT-D	1250	5.044	0.068	1427.992	20	117.895	0.026
	1350	5.372	0.030	7090.06	137.333	115.407	0.018
	1450	5.538	0.026	6264.774	117	117.894	0.012
BCZT-AB	1250	5.670	0.265	6864.922	109	117.668	0.019
	1350	5.192	0.058	7333.342	110.333	113.161	0.024
	1450	5.484	0.012	7786.893	127.333	118.040	0.014
BCZT-AD	1250	5.513	0.022	5428.755	106	120.483	0.038
	1350	5.492	0.047	6696.423	51	105.720	0.028
	1450	5.420	0.002	7181.887	41	106.765	0.035
BCZT-CD	1250	5.551	0.040	5493.425	32.333	107.014	0.021
	1350	5.286	0.030	6175.247	39.667	108.882	0.018
	1450	5.513	0.063	7282.937	93	110.826	0.027
BCZT-CB	1250	5.482	0.033	3774.391	33	118.810	0.082
	1350	5.481	0.009	5486.006	65.333	114.875	0.047
	1450	5.355	0.009	7124.909	58.333	111.499	0.042

**Table 3:** Selected properties of conventional lead-free and lead-based piezoelectrics.

Properties	Conventional Lead-Free	Lead-Based	V-Doped Samples
d_33_ [PCN^−1^]	Up to 200	Between 200 and 530	Between 20 and 137
Curie temperature [°C]	Between RT and 500	Between 200 and 450	Between 105 and 120

## Data Availability

Data will be made available upon request.
